# BIDIRECTIONAL ASSOCIATIONS BETWEEN SLEEP COMPLAINTS AND DEPRESSION: FINDINGS FROM THE NHATS STUDY

**DOI:** 10.1093/geroni/igz038.1340

**Published:** 2019-11-08

**Authors:** Minhui Liu, Sarah L Szanton, Michael V Vitiello

**Affiliations:** 1 Johns Hopkins University School of Nursing, Baltimore, Maryland, United States; 2 Johns Hopkins School of Nursing, Baltimore, Maryland, United States; 3 University of Washington, Seattle, Washington, United States

## Abstract

Depression and insomnia are prevalent in older adults and show bidirectional relationships. Sleep initiating and maintenance difficulties are the two frequently seen complaints of insomnia diagnostic criteria. Whether these two sleep complaints differ in their associations with depression is unknown. Using the National Health and Aging Trends Study (NHATS), we examined whether sleep initiating and maintenance difficulties at baseline (T1) predicted depression onset at 12 months (T2) and 24 months (T3) in 4,048 T1 non-depressed participants and whether depression at T1 predicted these two sleep complaints at T2 and T3 in 3,581 T1 non-insomnia participants. Participants who developed depression at T2 tended to be Hispanic, non-Hispanic black, less educated, live alone, physically inactive, and have more painful locations and chronic conditions. Participants with sleep complaints at T2 tended to be less educated, live alone, physically inactive, and have more painful locations and chronic conditions. Sleep initiating difficulty persistently predicted depression onset at T2 (OR: 1.62, 95% CI: 1.14, 2.31) and T3 (OR: 1.84, 95% CI: 1.21, 2.81) after adjusting demographics, lifestyles and health condition-related covariates. Depression at T1 persistently predicted sleep initiating difficulty at T2 (RRR: 2.19, 95% CI: 1.44, 3.34) and T3 (RRR: 1.70, 95% CI: 1.07, 2.70) after adjustment. Sleep maintenance difficulty at T1 did not predict depression onset at either time point and vice versa. This study suggests a bidirectional association of depression with sleep initiating difficulty but not sleep maintenance difficulty in older adults. Interventions targeting difficulty initiating sleep may moderate depression onset in older adults.

